# Cloning and characterization of an *Echinococcus granulosus* ecdysteroid hormone nuclear receptor HR3-like gene

**DOI:** 10.1051/parasite/2017037

**Published:** 2017-10-03

**Authors:** Mei Yang, Jun Li, Jun Wu, Hui Wang, Baoping Guo, Chuanchuan Wu, Xi Shou, Ning Yang, Zhuangzhi Zhang, Donald P. McManus, Fuchun Zhang, Wenbao Zhang

**Affiliations:** 1 Xinjiang Key Laboratory of Biological Resources and Genetic Engineering, College of Life Science and Technology, Xinjiang University, 14 Shengli Road, Urumqi 830046 PR China; 2 State Key Laboratory of Pathogenesis, Prevention and Treatment of Central Asian High Incidence Diseases, The First Affiliated Hospital of Xinjiang Medical University, Urumqi 830011 PR China; 3 Basic Medical College of Xinjiang Medical University, Urumqi 830011 PR China; 4 Public Health College of Xinjiang Medical University, Urumqi 830011 PR China; 5 Molecular Parasitology Laboratory, QIMR Berghofer, Herston, QLD, 4006 Australia; 6 Veterinary Research Institute, Xinjiang Academy of Animal Sciences, Urumqi 830000 PR China

**Keywords:** *Echinococcus granulosus*, hormone receptor 3, cloning, siRNA

## Abstract

Cystic echinococcosis is an important parasitic zoonosis caused by the dog tapeworm *Echinococcus granulosus*. Little is known about adult worm development at the molecular level. Transcription analysis showed that the *E. granulosus* hormone receptor 3-like (*EgHR3*) gene was expressed in protoscoleces and adult worms, indicating its role in early adult development. In this study, we cloned and characterized *EgHR3* showing that its cDNA contains an open reading frame (ORF) of 1890 bp encoding a 629 amino acid protein, which has a DNA-binding domain (DBD) and a ligand-binding domain (LBD). Immunolocalization revealed the protein was localized in the parenchyma of protoscoleces and adult worms. Real-time PCR analysis showed that *EgHR3* was expressed significantly more in adults than in other stages of development (*p*<0.01) and that its expression was especially high in the early stage of adult worm development induced by bile acids. *EgHR3* siRNA silenced 69–78% of the level of transcription in protoscoleces, which resulted in killing 43.6–60.9% of protoscoleces after 10 days of cultivation *in vitro*. *EgHR3* may play an essential role in early adult worm development and in maintaining adult biological processes and may represent a novel drug or vaccine target against echinococcosis.

## Abstract (fr)

L'échinococcose cystique est une importante zoonose parasitaire causée par le ténia du chien *Echinococcus granulosus*. Le développement des vers adultes est peu connu au niveau moléculaire. L'analyse de la transcription a montré que le gène de type 3 du récepteur de l'hormone (*EgHR3*) d'*E. granulosus* est exprimé dans les protoscolex et les vers adultes, indiquant son rôle dans le développement précoce des adultes. Dans cette étude, nous avons cloné et caractérisé *EgHR3* montrant que son ADNc contient un cadre de lecture ouvert (ORF) de 1890 pb codant pour une protéine de 629 acides aminés, qui a un domaine de liaison à l'ADN (DBD) et un domaine de liaison au ligand (LBD). L'immunolocalisation a révélé que la protéine était localisée dans le parenchyme des protoscolex et des vers adultes. L'analyse par PCR en temps réel a montré que *EgHR3* est exprimé beaucoup plus chez les adultes que dans d'autres stades de développement (*p*<0,01) et que son expression était particulièrement élevée au début du développement du ver adulte induit par les acides biliaires. Le siRNA *EgHR3* a réduit au silence 69 à 78 % du taux de transcription dans les protoscolex, ce qui a entraîné la destruction de 43,6 à 60,9 % des protoscolex après 10 jours de culture *in vitro*. *EgHR3* joue probablement un rôle essentiel dans le développement précoce des vers adultes et dans le maintien des processus biologiques des adultes et pourrait représenter une nouvelle cible pour des médicaments ou vaccins contre l'échinococcose.

## Introduction

Cystic echinococcosis (CE) is characterized by the presence of hydatid cysts in the liver, lungs or other organs in humans and herbivores. The hydatid cyst is the larval stage of the dog tapeworm *Echinococcus granulosus.* CE has a near-cosmopolitan distribution [1] and is a medically important disease causing substantial economic losses in endemic communities [2–4]. Globally, 3 million people have detectable hydatid cysts [5,6]. CE is highly endemic in western China with prevalence ranging from 2–12% [7].

The life cycle of *E. granulosus* is complex, involving two mammals, including an intermediate host, usually a domestic or wild ungulate (humans are accidental hosts), and a canine definitive host (dogs, wolves). Canines are infected by ingestion of animal offal harboring hydatid cysts containing protoscoleces (PSCs). After ingestion, PSCs evaginate, attach to the canine intestinal mucosa, and develop into adult worms  [3]. One remarkable feature of the PSC is its bidirectional development to either an adult worm (via sexual development) in the dog digestive tract, or a hydatid cyst (via asexual development) in the intermediate (or human) host. Smyth et al. showed that dog bile plays a crucial role in adult worm development and sexual differentiation  [8–10]. However, the molecular mechanisms underpinning these processes remain unknown.

Our previous transcription analysis showed that the *E. granulosus* hormone receptor 3-like (*EgHR*3) gene was expressed in PSCs and adult worms [11], suggesting a role in early adult development. A nuclear receptor (NR) superfamily 1F member-Hormone receptor 3 (HR3), is induced after puparium formation, represses early gene E75 expression and is a direct activator of the pre-pupal regulator FTZ-F1 (HR39) in insects [12,13]. HR3 is a major component of the ecdysteroid signaling pathway and mediates certain development-specific responses to ecdysteroid pulses [14].

Ecdysteroids (steroidal molting hormones of insects) have been detected and characterized in several non-arthropod phyla animals including coelenterate nematodes and Platyhelminthes [15], such as in blood fluke *Schistosoma mansoni* [16,17], the tapeworms *Moniezia expansa* [18] and *E. granulosus* [19]. Ecdysteroids function by binding to their receptors and regulate transcriptional activity of nuclear receptors including hormone receptor (HR)3 in insects. Studies suggested that these gene homologues may play roles in strobilization, the detachment of mature proglottids, vitellogenesis and embryogenesis in tapeworms [18,19].

In this study, we cloned and characterized the *E. granulosus* *HR3*-like (*EgHR3*) gene and showed that *EgHR3* is highly expressed in adult worms including early, immature adult worms, induced by bile acids. Silencing the *EgHR3* gene significantly impacted the survival of PSCs, indicating that EgHR3 may play an important role in the differentiation of PSC into adult worms.

## Methods

### Ethics statement

Dogs were used to raise adult worms of *E. granulosus* sensu stricto using methods previously described [3,20]. BALB/c mice were used to prepare anti-serum. The “Guidelines for the Care of Laboratory Animals” issued by the Ministry of Science and Technology of the People's Republic of China (2006) were strictly followed for the use of these animals. The dogs were maintained uncaged in a special facility at the Veterinary Research Institute of the Xinjiang Academy of Animal Science. They were provided with water and standard chow pellets. The strict protocols for the use of dogs and mice were approved by the Ethics Committee of the First Affiliated Hospital of Xinjiang Medical University (approval number IACUC-20120625003).

### Sample preparation

Fresh *E. granulosus* sensu stricto (*E. granulosus*) protoscoleces (PSCs) were directly aspirated from hydatid cysts present in the livers of sheep from a slaughterhouse in Changji, Xinjiang, China. The PSCs were washed 6 times with phosphate-buffered saline (PBS) before use.

Freshly obtained *E. granulosus* adult worms were collected from humanely euthanized dogs 35 days post-infection with PSCs, as described [20]. The worms were washed 10 times with PBS and directly stored at −80 °C or soaked in RNAlater (Sigma, Saint Louis, MO, USA) and stored at −80 °C until use.

### Total RNA extraction and cDNA synthesis

PSCs (100 μL) were used to extract total RNA using 1 mL TRIzol Reagent (Invitrogen, Carlsbad, CA, USA) and total RNA was extracted according to the manufacturer's instructions and details based on our previous study [21]. Samples were treated with RNase-free DNase I (Thermo Fisher Scientific, Waltham, MA, USA) for 30 min at 37 °C, to remove possible genomic DNA contamination. First, strand cDNA was synthesized using 100 ng of total RNA with a RevertAid reverse transcriptase kit (Thermo Fisher Scientific, Waltham, MA, USA) using Oligo (dT)18 (0.5 mg/mL) as the anchor primer. The final cDNA product was diluted 5-fold with nuclease-free water prior to its use in PCR analysis [22].

### EgHR3 cloning and expression

In previous genome studies [11,23], two genes (GenBank accession No. EUB60779.1 and CDS18863.1) encoding the DBD and LBD sequences of the HR3-like protein (EgHR3) in *E. granulosus* were predicted. Gene alignment analysis showed these two predicted fragments may come from the *EgHR*3 gene. To amplify full-length *EgHR3*, a pair of primers was designed (EgHR3-F: 5'ATGCTATGTCTCGTATGCGGAGACA3' and EgHR3-R: 5' CTAGACAAGAGAAAAGGTTTCGCTATACA3') and the full-length of cDNA was amplified by PCR. To express the DNA binding domain (DBD) of the protein, two primers containing *Bam*H I and *Not* I (italicized letters) restriction sites were designed and cloned into the pET 30 vector: *EgHR3*-DBD-F: 5'cgc*GGATCC* ATGCTATGTCTCGTATGCGGAGACACT3' and *EgHR3*-DBD-R: 5'ataagaat*GCGGCCGC*CTATTTAGATATCTTTTTAGCGCTACATCTGCC3'. The amplified PCR products were digested with restriction enzymes and cloned into the expression vector pET-30a(+) (Invitrogen) with a 6 histidine (His) fusion tag. The inserted sequence was verified by DNA sequencing. The resulting plasmid was transformed into *E. coli* BL21 (DE3) cells. The expression of *EgHR3-DBD* was induced with 0.8 mM isopropyl-β-D-thiogalactopyranoside (IPTG) at 22 °C for 20 h. The recombinant fusion protein (rEgHR3*-*DBD) was purified using a His tagged affinity column (Invitrogen). Polyclonal antiserum against rEgHR3*-*DBD was generated in BALB/c mice. Each mouse was subcutaneously immunized with 25 µg of the purified recombinant protein emulsified with complete Freund's adjuvant as primary immunization. This was followed by 2 subcutaneous injections of rEgHR3*-*DBD and 2 intraperitoneal injections as boosts with the same dose of rEgHR3*-*DBD emulsified with incomplete Freund's adjuvant with a two-week interval between injections. Blood was collected for serum preparation one week after the final immunization. The serotitre of the antiserum against the recombinant protein was determined by enzyme-linked immunosorbent assay (ELISA).

Western blot analysis was preformed according to our previous study [21].

### Immunoblotting for probing *E. granulosus* native proteins

To extract native proteins, parasite tissues (PSCs, adult worms and cyst germinal layers) of *E. granulosus* were collected. *E. granulosus* cyst germinal layers were prepared according to our previous study [11]. Parasite tissue was suspended in lysis buffer (Beyotime, Shanghai, China) containing 1 mM PMSF (Sigma), homogenized using a homogenizer for 5 min, and then sonicated on ice until the suspension became clear. The homogenate was centrifuged at 12 000 rpm at 4 °C for 60 min and the supernatant containing soluble proteins was retained. To extract insoluble proteins, the tissue pellets were re-suspended with lysis buffer containing 1% (w/v) SDS, and heated at 60 °C for 20 min.

Native *E. granulosus* proteins were separated through 12% SDS-PAGE gels and Western blot analysis was used to identify the parasite proteins.

### Mass spectrometry of rEgHR3-DBD

To determine whether rEgHR3*-*DBD was the correct target protein, the recombinant polypeptide was electrophoresed on a 12% SDS-PAGE gel and then stained with Coomassie Blue. The target band putatively containing rEgHR3*-*DBD was cut and gel strips were sent to the Beijing Genomics Institute (BGI, Shenzhen) for proteomic sequencing analysis.

### Immunofluorescence analysis of EgHR3 in adults and PSCs of *E. granulosus*

PSCs and adult worms of *E. granulosus* were separately fixed in 4% (v/v) paraformaldehyde buffered in PBS. The fixed parasites were dehydrated in ethanol solution, embedded in paraffin and sectioned. The sections were probed with the affinity-purified mouse anti-rEgHR3-DBD serum and conjugated goat anti-mouse fluorescent antibody-CF^TM^568. After rinsing four times with PBST (5 min each) the slide sections were stained with the fluorescent nuclear stain DAPI for 10 min, and then rinsed a further four times with PBST. The slides were then imaged using a Leica TCS SP8 Confocal laser scanning microscope.

### Bioinformatics analysis of EgHR3-like

The physicochemical properties of EgHR3 were predicted by the ProtParam tool (http://www.expasy.ch/tools/protparam.html). Functional domains and motif sites were analyzed by Motifscan (http://myhits.isb-sib.ch/cgi-bin/motif_scan). BioEdit was used to analyze the homology of the DBD and LBD in the target sequence. Data for all orthologous proteins were collected using Blast explorer according to the highest scores among the top 100 hits. A phylogenetic tree of the DBD of the protein family was constructed using the MEGA6.0 neighbor-joining algorithm and the Maximum Likelihood (ML) method (1000 replicates) [24]. All positions containing gaps and missing data were eliminated. Evolutionary distances were computed by the Poisson correction method.

### Quantitative real-time PCR analysis of EgHR3-like gene expression

*E. granulosus* PSCs were washed 6 times with 1×PBS (PSC sample), treated with pepsin and trypsin and cultured for 3 hours (PSC-3h) to 2 weeks (PSC-2w sample) in Smyth's published culture system [8,9] with or without sodium taurocholate in parallel.

Total RNA was extracted from PSCs, cultured cyst germinal layer (CM) [11], and 35 day adult worms [20] of *E. granulosus* using TRIZOL (Invitrogen) according to the manufacturer's instructions. A RevertAid reverse transcriptase kit (Thermo Fisher Scientific) was then used to synthesize cDNA. The sense and antisense primers for *EgHR3-like* were 5'-TGGCAGCGACACTACCTTTA-3', 5'-AGTTCGCCTTGTTTCCCTTG-3', and primers for *Eg-eif* (*E. granulosus* eukaryotic translation initiation factor) as an internal control [25] were 5'- GGGTAGAGAAATACATGCCATTG-3' and 5'-TTCATCACTAACAGCGGAAGG-3'. The conditions used for real-time PCR were 95 °C for 15 min, 95 °C for 15 s, 60 °C for 30 s, and 72 °C for 30 s for 40 cycles; the reaction was terminated by cooling to 4 °C. All samples were run in triplicate. A SYBR Green PCR Kit (Qiagen, Germany) was used for the PCR and the reaction was conducted in an iCycler iQ™ system (BIO-RAD), with data analyzed according to the 2^−ΔΔCt^ method [26]. The efficiency of the primers was tested and it was confirmed that no additional products are amplified. Statistical analysis used one-way ANOVA and Tukey's multiple comparison tests.

### RNA interference (RNAi) assay

PSCs were treated with pepsin and cultured in RPMI1640 medium containing fetal calf serum using the same incubation protocols as described above. Negative control RNAi experiments were performed using either fluorescently labeled (Cy3-labeled negative control siRNA, Ribobio, Guangzhou, China) or non-labeled negative control small RNA (Silencer negative control siRNA, Ribobio). Control RNAs without targets to any human, mouse, rat or *E. granulosus* genes were selected.

For electroporation, 100 μL electroporation buffer containing approximately 2000 PSCs was placed in a 4 mm cuvette, and siRNA was added to give final concentrations of 1 μM or 5 μM. Pilot experiments indicated optimum electroporation occurred at 125 V–20 ms (Gene pulser, Bio-Rad) for the introduction of siRNA into PSCs and this parameter was used in all subsequent experiments. After incubation at 37 °C for 10 min, the PSCs in buffer were transferred to 1 mL culture medium and were then further incubated at 37 °C in 24-well plates in the presence of 5% CO_2_ in the dark. After 30 min and 2 h of silencing treatment, the PSCs were washed with PBS and viewed under a Leica TCS SP8 Confocal laser scanning microscope to evaluate the efficacy of the treatment.

Four siRNA probes were used for the gene silencing analysis: non-labeled negative control siRNA (NsiRNA), siRNA1 (siRNA*EgHR3*-73), siRNA2 (siRNA*EgHR3*-304) and siRNA3 (siRNA*EgHR3*-373). The siRNA probes were 21 nt long, with 3' overhangs. The sequence of siRNA1 was 5'-GGAUUCUUUAGACGGGCAUTT-3'; the sequence of the siRNA2 was 5'-CCUGAGGACAGCAACCAAUTT-3'; and the sequence of the siRNA3 was 5'-CCAGGCACGAAUCUGUCAUTT-3'. After electroporation, the PSCs were incubated at 37 °C for 10 min in RPMI1640 medium containing fetal calf serum and antibiotics, like above. RNAi effects on mRNA levels were evaluated using real-time PCR [27]. Each relative amount was normalized to the untreated control at day 0, and the data were analyzed according to the 2^−ΔΔCt^ method. Statistical analysis was then performed by one-way ANOVA and Tukey's multiple comparison test.

The effects of siRNA (5 µM) introduction by electroporation on the viability of PSCs were evaluated in all cultured samples on days 3, 6, 10 and 15. Viability (%) was calculated by staining with 0.1% methylene blue [28] and counting the number of live PSCs that were clear in appearance and contained transparent structures; dead PSCs appeared opaque and had a rough surface and damaged inner structures. All samples were run in triplicate.

## Results

### Cloning and sequence analysis of the EgHR3-like gene

**Figure 1:**
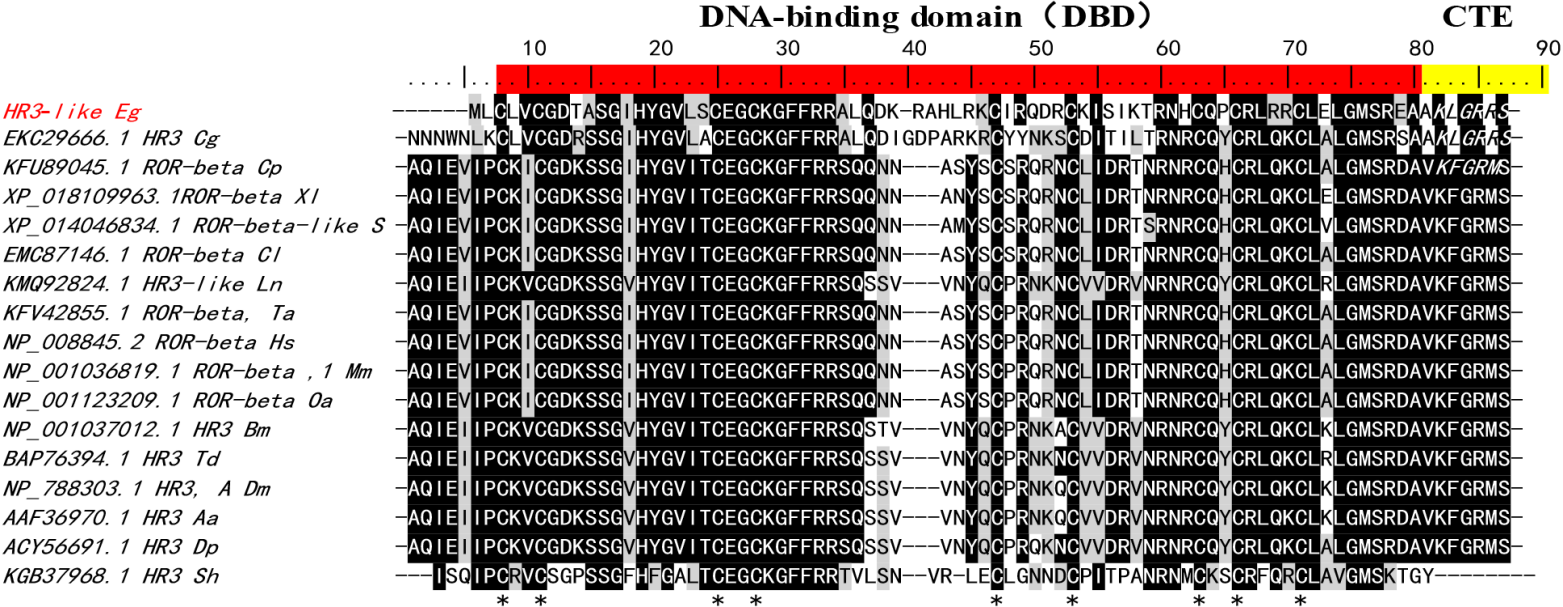
Sequence alignment of the DNA-binding domain (DBD) and its C-terminal extension (CTE**)** in the *E. granulosus* EgHR3-like protein (ART84255.1) with homologs from other species. The conserved residues of DBD are highlighted in red. The conserved residues of CTE are highlighted in yellow. The conserved 8 cysteines of two C4-type zinc fingers are indicated with ^*^. The GRIP-box in CTE is indicated by amino acid residues KXGRZS in italics. Sequences used for the alignment are shown in Table 1.

Sequence analysis showed that two predicted gene fragments of *E. granulosus* (GenBank accession No. EUB60779.1 and CDS18863.1) [11,23] comprise the full-length gene of *EgHR3*. Based on the sequence, we designed primers to amplify the cDNA sequence of the *EgHR3*-like gene by PCR. The full-length cDNA obtained was identical in sequence to the two predicted sequences published recently [11,23] (data not shown). The cDNA has an open reading frame (ORF) of 1890 bp encoding 629 amino acids with a predicted molecular mass (MW) of 70 kDa and a predicted isoelectric point (PI) of 9.09. Sequence analysis using BLAST and domain sequence alignment of *EgHR3*, with homologous sequences from other species, showed that the amplified sequence contains a characteristic and conserved DNA-binding domain (DBD) associated with nuclear receptors (NR). The DBD is composed of two C4-type zinc fingers containing 8 conserved cysteines (Figure 1). Residues 3–68 represent a zinc binding region. Motif scan analysis confirmed DBD at residues 3 to 74. In addition, the alignment showed the first zinc finger contains two conserved sequence regions (“CGD” and “CEGCKGFFRR”) (Figure 1). These conserved sequences may form the core for binding DNA. The DBD of *EgHR3* is equipped with two zinc finger motifs and an adjacent GRIP-box [14] with a “KLGRRS” sequence at the C-terminal extension (CTE) to the DBD (Figure 1).

**Figure 2:**
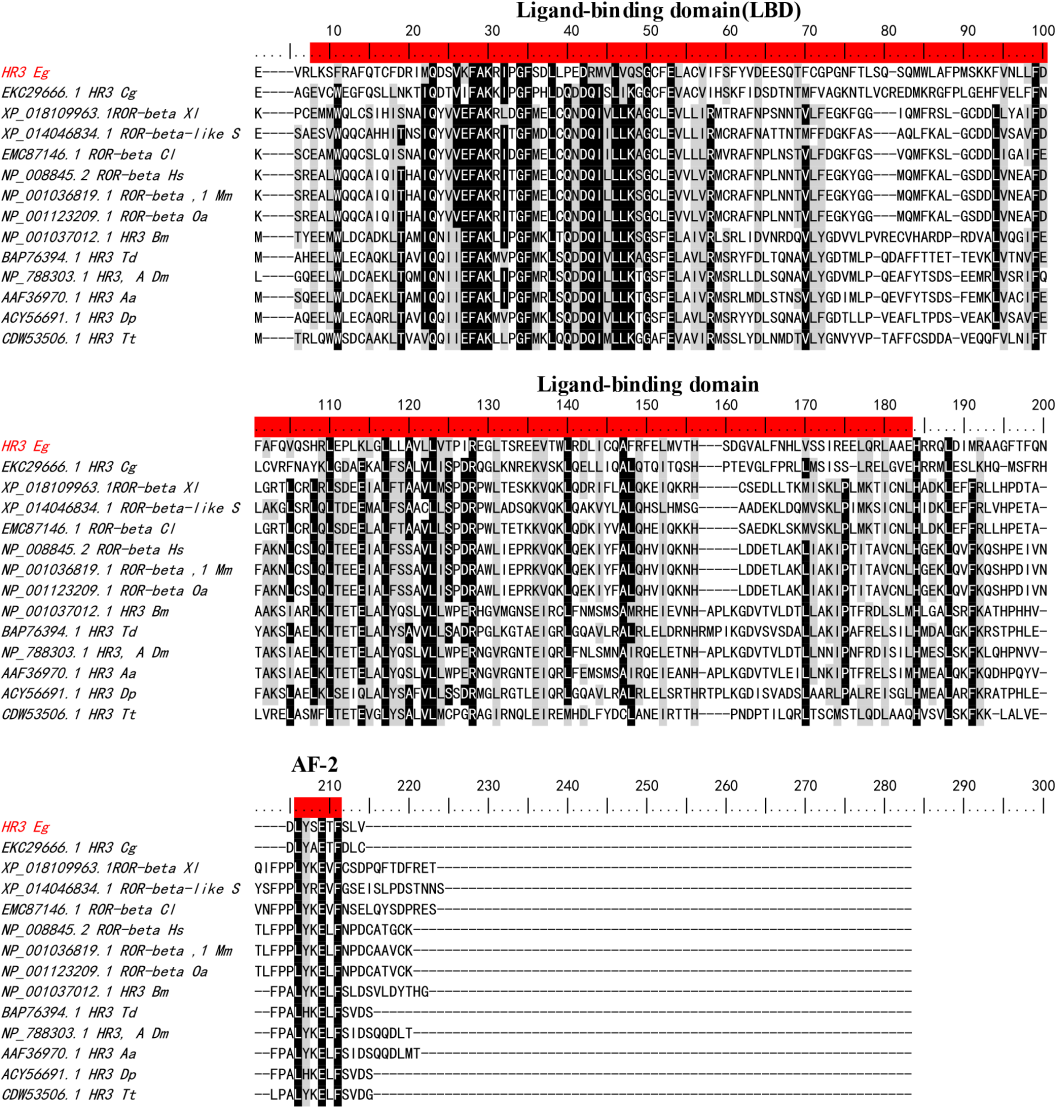
Sequence alignment of the ligand-binding domain (LBD) of *E. granulosus* EgHR3-like protein with the LBD from other species. The two conserved motifs and the putative autonomous activation domain (AF-2) are highlighted in red. Sequences used for alignment are shown in Table 1.

*EgHR3* also contains a moderately conserved ligand-binding domain (LBD) and a putative ligand-dependent activation function domain 2 (AF-2) (Figure 2) with a conserved “LYSETF” sequence.

### Phylogenetic analysis

**Figure 3:**
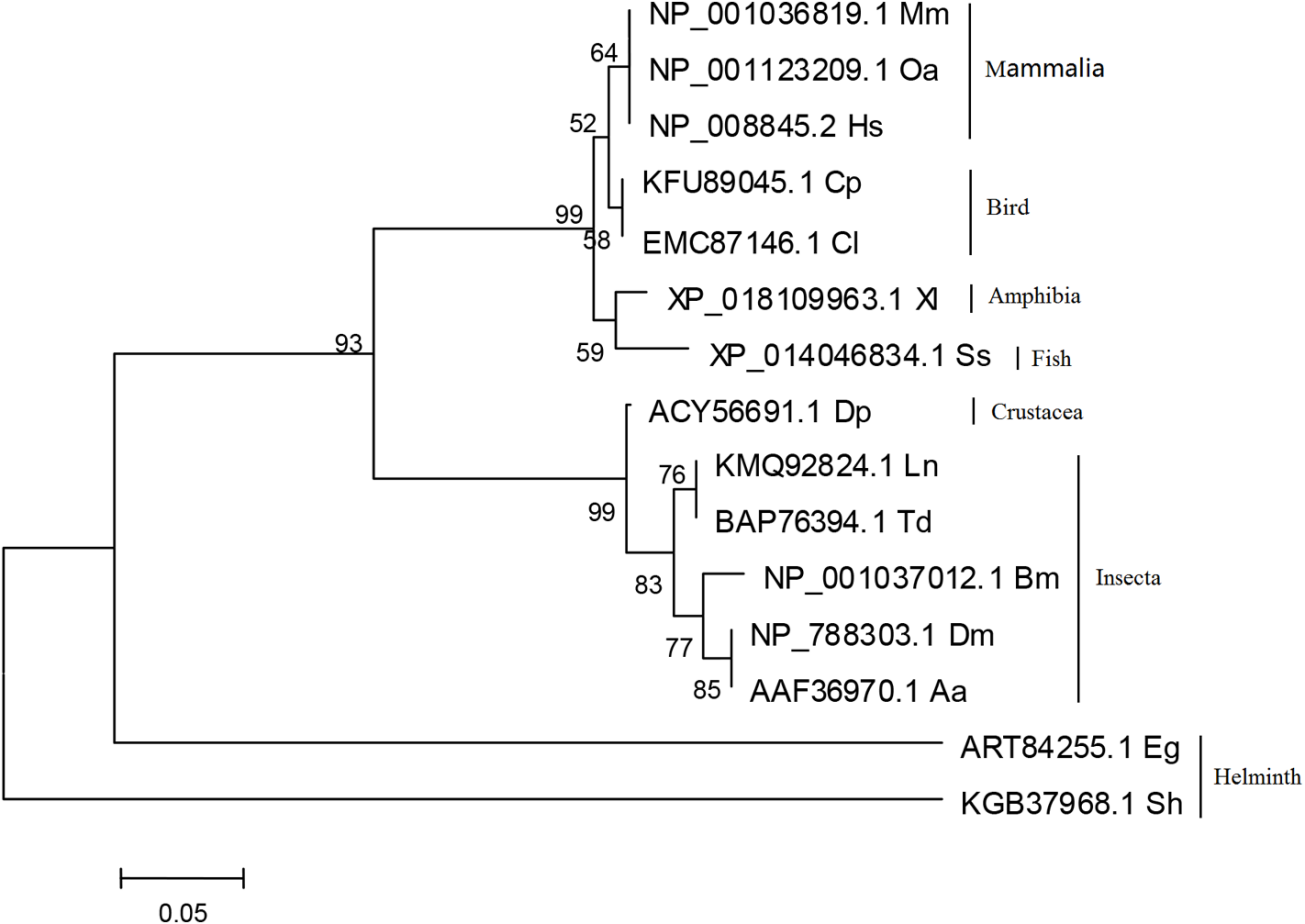
Phylogenetic analysis of the DBD sequence of *E. granulosus* EgHR3-like protein (ART84255.1) with DBD sequences of HR3/ROR from other species. Sequences used for phylogenetic analysis are shown in Table 1.

To further determine the evolutionary relationships of *EgHR3*, we compared the DNA binding domain (DBD) of *EgHR3* with this region in 14 other species including humans (*Homo sapiens*, *Hs*), sheep (*Ovis aries*, *Oa*), mouse (*Mus musculus*, *Mm*), bird (*Chaetura pelagica, Cp; Columba livia, Cl*), frog (*Xenopus laevis*, *Xl*), fish (*Salmo salar, Ss*), silkworm (*Bombyx mori*, *Bm*), fruit fly (*Drosophila melanogaster*, *Dm*) and worm (*Schistosoma haematobium*, *Sh*) (Table 1). The phylogenetic analysis revealed an evolution relationship from mammalian species (human and sheep) to invertebrate species (worms) (Figure 3). The sequences of helminths including *S. haematobium*, and *E. granulosus* formed separate and distinct clades.

**Table 1:** Sequences used for alignment and phylogenetic analysis.

Protein ID	Protein names	Species (abbreviation)	Phyla	Nomenclature
ART84255.1	HR3-like	*E. granulosus* (*Eg*)	Platyhelminthes	NR1F
KGB37968.1	HR3	*Schistosoma haematobium* (*Sh*)	Platyhelminthes	NR1F
EKC29666.1	HR3	*Crassostrea gigas* (*Cg*)	Mollusca	NR1F
NP_001037012.1	HR3	*Bombyx mori* (*Bm*)	Arthropoda (Insecta)	NR1F
KMQ92824.1	HR3-like	*Lasius niger* (*Ln*)	Arthropoda (Insecta)	NR1F
AAF36970.1	HR3	*Aedes aegypti* (*Aa*)	Arthropoda (Insecta)	NR1F
ACY56691.1	HR3	*Daphnia pulex* (*Dp*)	Arthropoda (Crustacea)	NR1F
BAP76394.1	HR3	*Thermobia domestica* (*Td*)	Arthropoda (Insecta)	NR1F
NP_788303.1	HR3 isoform A	*Drosophila melanogaster* (*Dm*)	Arthropoda (Insecta)	NR1F
XP_014046834.1	ROR-beta	*Salmo salar* (*Ss*)	Chordata (Osteichthyes)	NR1F
XP_018109963.1	ROR-beta-like	*Xenopus laevis* (*Xl*)	Chordata (Amphibia)	NR1F
EMC87146.1	ROR-beta, partial	*Columba livia* (*Cl*)	Chordata (Aves)	NR1F
KFV42855.1	ROR-beta, partial	*Tyto alba* (*Ta*)	Chordata (Aves)	NR1F
KFU89045.1	ROR-beta, partial	*Chaetura pelagica* (*Cp*)	Chordata (Aves)	NR1F
NP_001036819.1	ROR-beta isoform 1	*Mus musculus* (*Mm*)	Chordata (Mammalia)	NR1F
NP_001123209.1	ROR-beta	*Ovis aries* (*Oa*)	Chordata (Mammalia)	NR1F
NP_008845.2	ROR-beta	*Homo sapiens* (*Hs*)	Chordata (Mammalia)	NR1F
CDW53506.1	HR3	*Trichuris trichiura* (*Tt*)	Nematoda	NR1F

### Expression and purification of rEgHR3-DBD

**Figure 4:**
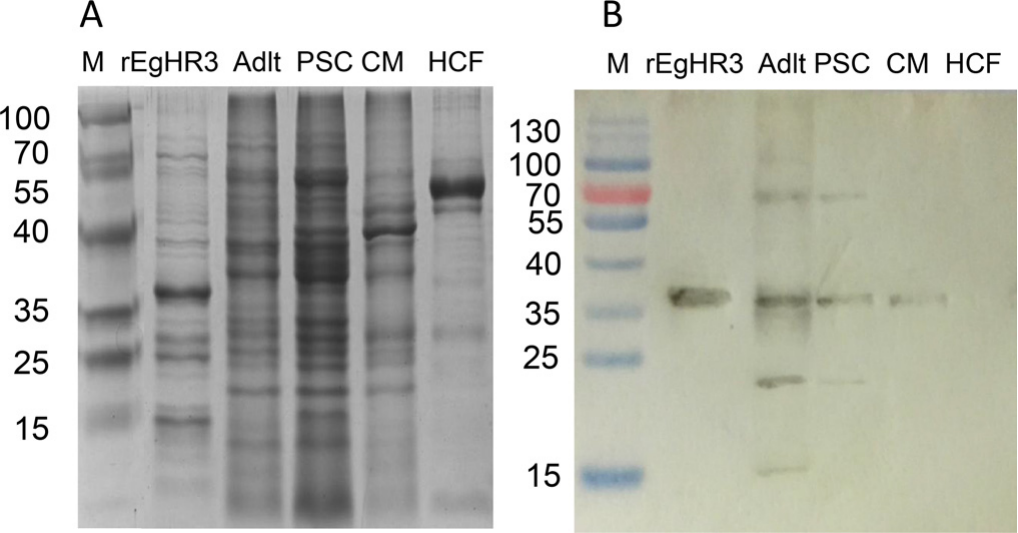
SDS-PAGE and Western blot analysis of EgHR3-like protein. SDS-PAGE analysis of recombinant His-EgHR3 (rEgHR3), *E. granulosus* soluble protein extracts from adult worms (Adult), protoscoleces (PSCs), cyst germinal layer membranes (CMs) and hydatid cyst fluid (HCF) (A). Western Blot analysis (using mouse anti-rEgHR3 antiserum). Protein markers are indicated in kDa (B).

*EgHR3-DBD* was successfully cloned into a pET vector and expressed in *E. coli* following IPTG induction. Figure 4A shows the yield and size of the expressed protein on SDS-PAGE. The molecular mass of the affinity purified protein with His tag is about 37 kDa, which is consistent with the predicted size by bioinformatics analysis. A band corresponding to the expected EgHR3-DBD was present in the sample of the supernatant of cell lysates, indicating that the receptor protein is partially soluble. The purified His-tag protein was recognized by an anti-His tag primary antibody in Western blot analysis (data not shown). To confirm that the target EgHR3-DBD protein was expressed, we electrophoresed the recombinant protein purified by SDS-PAGE, stained and excised the band, submitted it to BGI-Shenzhen for protein sequencing and the proteomic analysis confirmed the identity of the target protein sequence (data not shown).

### Western blot analysis and immunolocalization of native EgHR3

Antiserum produced in mice against purified recombinant EgHR3-DBD induced an average serum titre of 1:500 000 determined by ELISA (data not shown). The antiserum was purified and used to probe native proteins isolated from different stages of *E. granulosus* by Western blot analysis. Figure 4B shows the native protein band, with a molecular size of approximately 70 kDa, in PSCs and adult worms. The protein is highly expressed in PSCs and adult worms, whereas no band was detected in hydatid cyst fluid. The Western blotting also showed an additional native protein band, with a molecular size of approximately 35 kDa in PSCs and adult worms, which may indicate partial degradation of the native protein.

**Figure 5:**
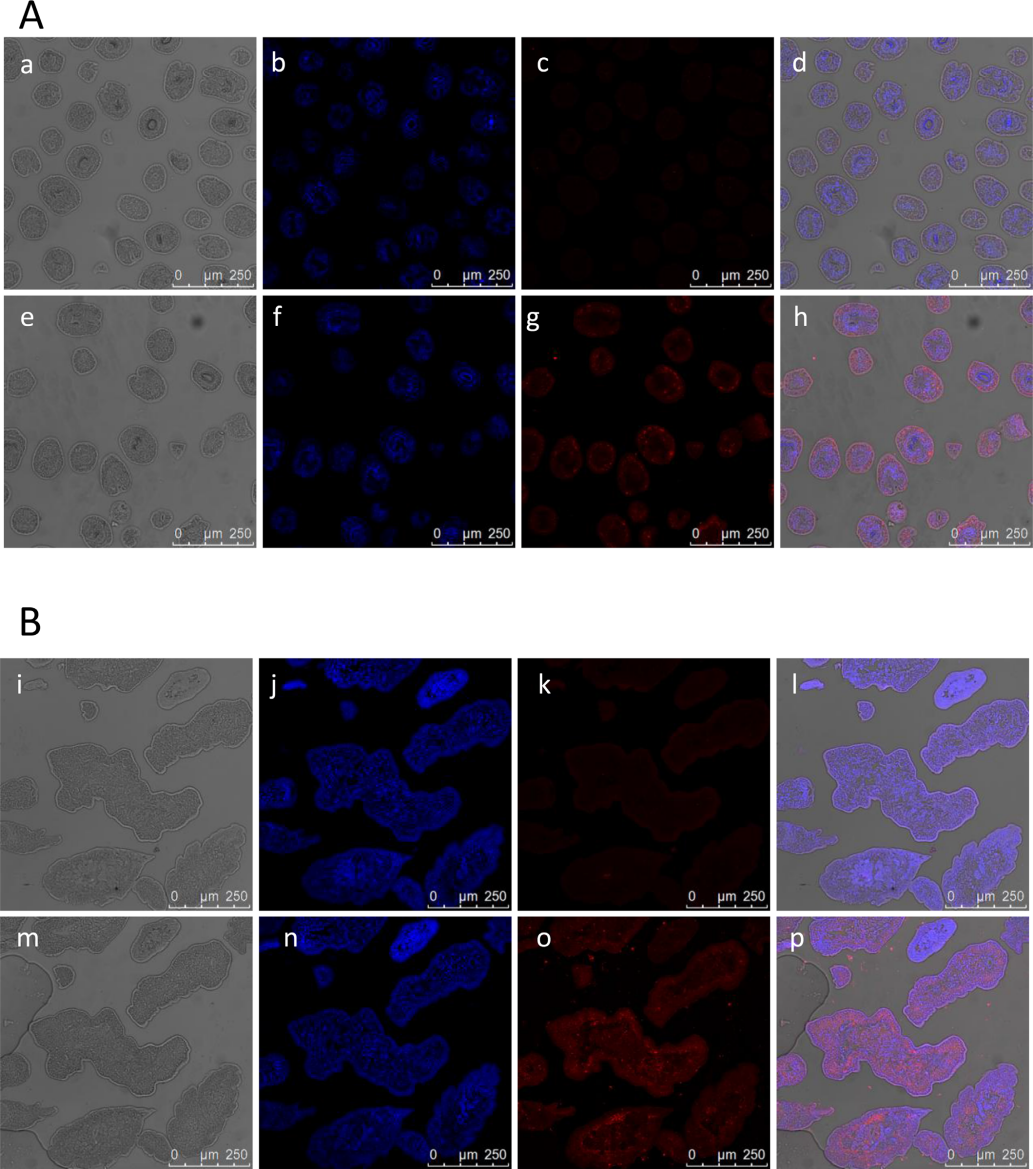
Immunolocalization of EgHR3 in protoscoleces (PSCs) and adult worms of *E. granulosus*. EgHR3 in PSCs: a and e, showing normal structure of PSC; b and f, stained with DAPI; c and g, probed with normal mouse serum and anti-EgHR3 antiserum, respectively; d and h, combined a, b and c, or e, f and g, respectively, showing co-localization of nucleus and EgHR3 (A). EgHR3 in adult worms of *E. granulosus*: i and m, showing normal structure of adult worms; j and n, stained with DAPI; k and o, probed with normal mouse serum and anti-EgHR3 antiserum, respectively; l and p, combined i, j and k, or m, n and o, respectively, showing co-localization of nuclei and EgHR3 (B).

We used the affinity-purified anti-EgHR3-DBD antiserum to probe parasite sections in situ by immunofluorescence, which revealed that the EgHR3 protein was evenly distributed throughout all tissues of both the adult worms and PSCs (Figure 5). No specific immunofluorescence was observed in either stage when probed with naive control serum (Figure 5).

### Transcription in different developmental stages of *E. granulosus*

**Figure 6:**
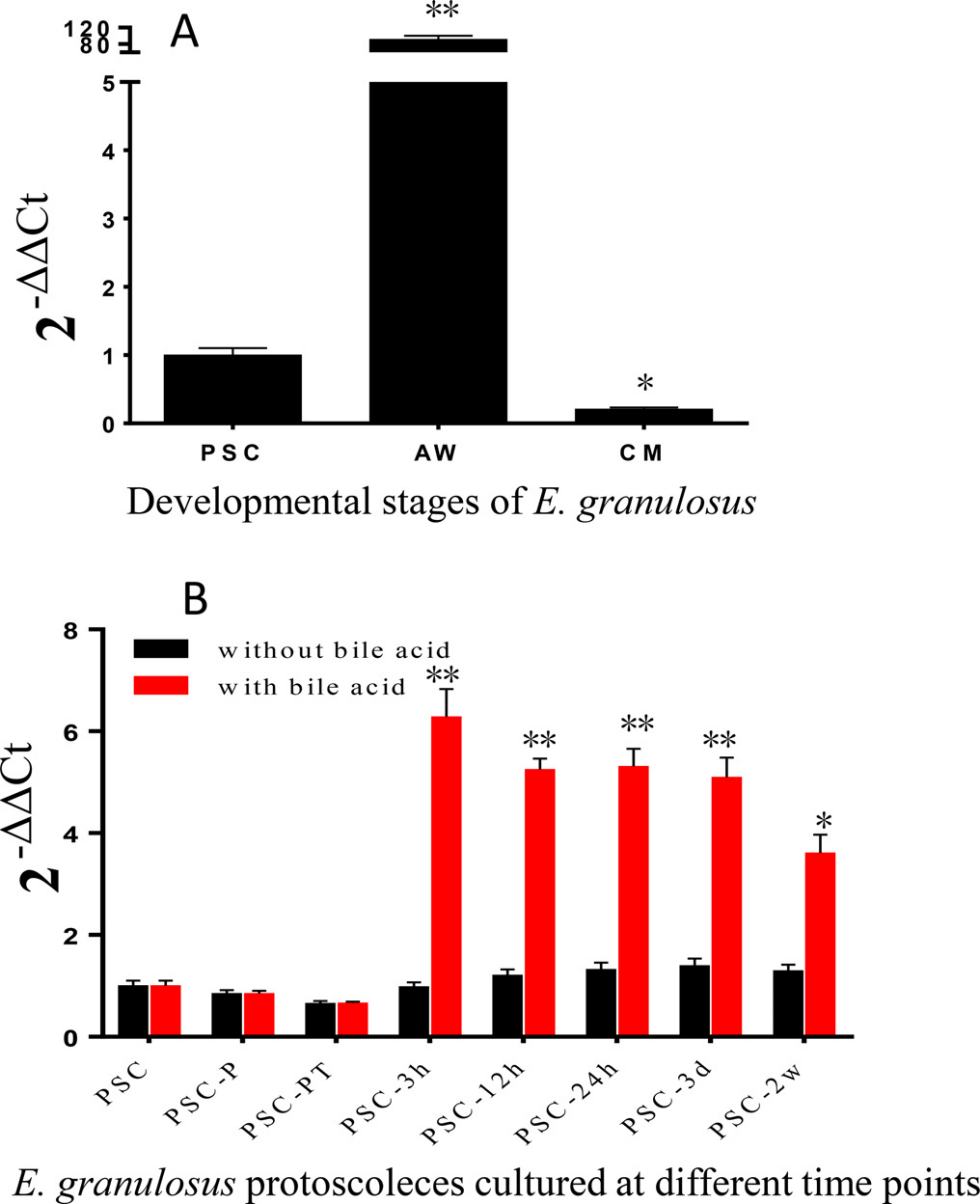
Quantitative real-time PCR analysis of the transcription of *EgHR3.* The transcription of *EgHR3* in different developmental stages of *E. granulosus*: AW, adult worms; CM, cyst germinal layer (A). The transcription of *EgHR3* at different time points of cultured *E. granulosus* protoscoleces (B). PSCs, protoscoleces; PSC-P: PSCs treated with pepsin; PSC-PT: PSCs treated with pepsin and trypsin; PSC-B3h, PSC-B12h, PSC-B24h: PSCs treated with pepsin and trypsin then cultured with sodium taurocholate for 3, 12, and 24 h; PSC-B3d: PSCs treated with pepsin and trypsin then cultured with sodium taurocholate for 3 days; PSC-B2w: PSCs treated with pepsin and trypsin then cultured with sodium taurocholate for 2 weeks. The data were normalized using the housekeeping gene *Eg-eif*. ^*^, significant difference compared with the expression level in PSCs.

To determine whether expression of the *EgHR3* is regulated by bile acids, we cultured PSCs in a medium containing the bile salt sodium taurocholate. In culture in the absence of bile acids, PSCs develop into cysts [8,9]. We used real-time PCR to determine the transcriptional levels of *EgHR3* in PSCs (cultured with or without sodium taurocholate), adult worms and cyst membranes. Transcription levels were normalized with the transcription of *Eg-eif* as a house-keeping gene [25]. Figure 6A shows that *EgHR3* was highly transcribed in adult worms. The transcription was 92- and 441-fold higher than in PSCs and cyst germinal layer membrane, respectively.

To further examine the expression of *EgHR3* in early adult worm development, we compared the gene expression in PSCs at different points with or without sodium taurocholate in the medium. The highest level of *EgHR3* transcription occurred 3 h after PSCs were cultured with sodium taurocholate (PSC-B3h); this was 6.3 times higher than in PSCs cultured in PBS (as a control PSC sample) or in PSCs cultured for 3 h without sodium taurocholate (Figure 6B). No statistical differences were observed in the gene expression of *EgHR3* in PSCs at different time points cultured in the absence of sodium taurocholate.

### RNAi silencing of the EgHR3 gene in *E. granulosus* PSCs

**Figure 7:**
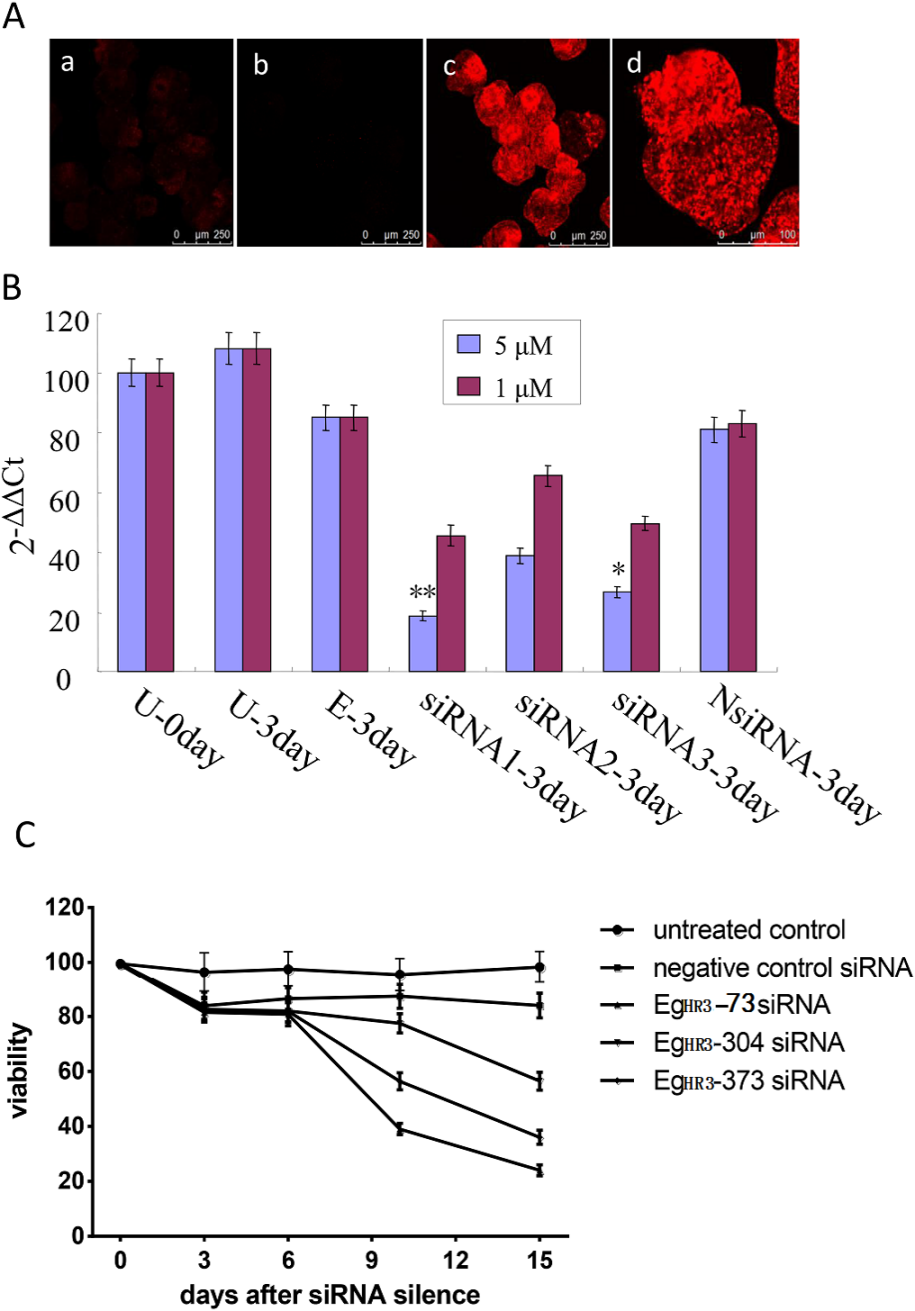
Silencing of *EgHR3* by siRNAs. Efficiency of electroporation for transformation of control labeled siRNA into PSC. a and b, untransformed PSC; c and d, transformed PSCs (A). Silencing efficacy of siRNA for *EgHR3* in PSCs at two concentrations (1 and 5 μM) of siRNA. U-0 day and U-3 day, untreated PSCs or untreated PSCs cultured in vitro for 3 days; E-3 day, PSCs treated by electroporation only and then cultured for 3 days; siRNA1-3, siRNA*EgHR3*-73, siRNA*EgHR3*-304 and siRNA*EgHR3*-373 transfected PSCs which were cultured in vitro for 3 days; NsiRNA-3 day, non-labeled negative control siRNA transfected PSCs which were cultured in vitro for 3 days (B). Silencing efficacy of small RNA fragments on the viability of *E. granulosus* PSC (C).

To determine whether silencing of *EgHR3* had an effect on parasite growth and development, we designed three small interfering RNA (siRNA) fragments based on the *EgHR3* sequence and introduced the siRNAs into PSCs. A pre-transformation assay using fluorescent dye labeled siRNA showed that PSCs could be highly and efficiently transfected with small RNA by electroporation at 125 V for 20 milliseconds (Figure 7A).

We then transfected the three siRNAs into PSCs in culture medium containing sodium taurocholate after the PSCs had been treated with acid pepsin. RT-PCR analysis revealed that, compared with PSC transfected with a negative control siRNA, the level of *EgHR3* transcription was reduced significantly (*p*<0.01) by 78% and 69% in PSCs transfected with *EgHR3*-73 and *EgHR3*-373, respectively, at a concentration of 5 μM siRNA after 3 days of culture (Figure 7B). No statistical differences were observed between the NsiRNA and siRNA*Eg HR3*-304, indicating siRNA*EgHR3-304* had no inhibitory effect. A lower concentration (1 μM) of the siRNAs did not significantly change the level of *EgHR3* transcription.

We stained and counted live PSCs at different time points after culture and calculated their viability as a measure of phenotypic change induced by the siRNA. There was no significant difference in viability on day 6 of any of the siRNA transfected PSCs compared with PSCs transfected with negative control siRNA (*p*>0.05). By day 10, however, there was 60.9% killing of PSCs transfected with siRNA*EgHR3-73* compared with PSCs transfected with control NsiRNA (*p*<0.01). The order of the killing effect (or decreased survival) was siRNA*EgHR3-73*>siRNA*EgHR3-373*>siRNA*EgHR3-304* (Figure 7C).

## Discussion

Nuclear receptors (NRs) are ligand-regulated transcription factors that share a common domain architecture containing a DNA binding domain (DBD) with a highly conserved zinc finger motif and a structurally conserved ligand binding domain (LBD) [29,30]. We isolated HR3 from *E. granulosus*, which is a member of the NR1F superfamily. Here, we identified the *E. granulosus* HR3-like gene (*EgHR3*) based on its conserved structure including two zinc finger motifs containing a group of 8 cysteine residues which form the core for binding the zinc ion. Beside the 8 cysteines, the conserved sequences “CGD” and “CEGCKGFFRR” are located in the first zinc finger (Figure 1) of all the sequences aligned. The short conserved sequences may represent motifs forming the core of the DNA binding sites. HR3 in *Drosophila melanogaster* also contains a highly conserved C-terminal extension (CTE) to the DBD [14]. This CTE contains a “GRIP-box” sequence, which has the consensus sequence (K/R)XGRZ(P/S), where X is any amino acid and Z represents a hydrophobic amino acid [14,31]. The GRIP-box typically provides stability to a monomeric receptor protein when binding DNA [32]. The EgHR3 DBD is equipped with two zinc finger motifs and a GRIP-box adjacent to the DBD; the GRIP-box sequence of EgHR3 is “KLGRRS” (Figure 1). EgHR3 also contains a moderately conserved LBD and a putative AF-2 domain core (Figure 2). The AF-2 consensus sequence of NR1F is LYXEZF, where X and Z are any amino acid. The AF-2 sequence of EgHR3 is “LYSETF” (Figure 2). All these features indicate that the isolated sequence from *E. granulosus* is an *HR3*-like gene. Phylogenetic analysis of EgHR3-DBD confirmed that the isolated gene from *E. granulosus* is actually an *HR3* (Figure 3). The phylogenetic analysis further revealed that all HR3-DBDs analyzed formed three distinct clusters with *S. haematobium* located in a distinct clade (Figure 3).

Ecdysteroids have been reported to present in schistosomes with varying levels during the development of the post-cercarial stages [16]. There is evidence that *S. mansoni* can synthesize ecdysone, which plays a role in stimulating growth and vitellogenesis [17]. In *S. mansoni* miracidia, β-ecdysterone was reported to be effective in stimulating host location behavior [33,34]. Ecdysone may play a role in strobilization (proglottid differentiation) in *Moniezia expansa* [18]. Ecdysteroids have also been identified in the PSCs of *E. granulosus* [19], suggesting that the role of ecdysone in molting in the Ecdysozoa lineage was acquired after the split of the Ecdysozoa and Lophotrochozoa. By analogy with the mode of action in insects, ecdysone and 20-OH ecdysone may play a key role in tapeworm development and differentiation, including strobilization, the detachment of mature proglottids, vitellogenesis and embryogenesis [19].

Ecdysone binding to its receptors directly regulates the transcriptional activity of the other 3 nuclear receptors, ecdysone-induced protein 75 (E75), HR3 and HR4. The previous genome studies showed that *Echinococcus* spp. had the protein molecules of the ecdysteroid signaling pathway, including E75 (GenBank: EUB64236.1), E78 (GenBank: CDS17388.1), FTZ-1(HR39) (GenBank: CDS15732.1) and HR3 [11,23]. In the study, we showed that *EgHR*3 was highly expressed in adult worms (more than 400 times higher than the cyst membrane), and significantly induced in PSCs in the medium containing bile acids, indicating that *EgHR*3 plays an important role in adult worms, especially in early adult worm development regulated by bile acids.

In insects, most effects of ecdysone are mediated through the heterodimeric Ecdysone receptor-Ultraspiracle receptor (ECR-USP) [12]. ECR is clearly the invertebrate ortholog of the farnesoid X receptor (FXR) of vertebrates with USP being the invertebrate ortholog of RXR [35,36]. E75 is induced as a primary early response gene, while HR3 and HR4 are induced as early-late genes [12]. HR3 is induced after puparium formation, and then down-regulates the early gene E75 [12,13].

**Figure 8:**
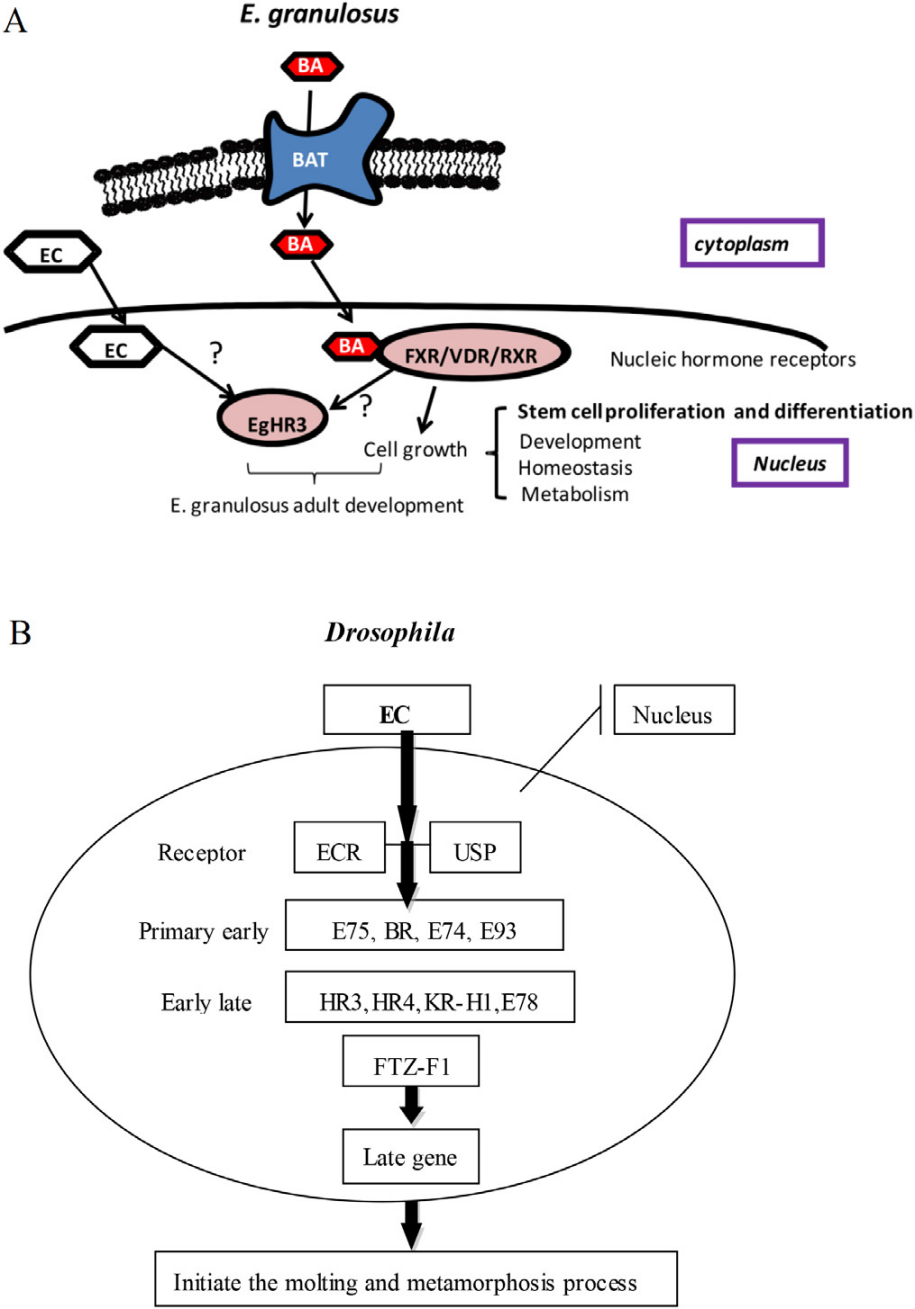
Summary of the ecdysone regulatory cascade. Summary of the putative regulatory cascade of *E. granulosus* adult worm development (A). Bile acid (BA) is transported into the cytoplasm, and is then transported into the nucleus where it binds FXR/DVR/RXR to stimulate cell proliferation and growth. The BA-FXR/RXR complex or EC (ecdysone) may also regulate the transcriptional activity of *EgHR3* to regulate adult worm development. Summary of the ecdysone regulatory cascade, with the 11 transcription factors known to act as classic early regulators during the onset of *Drosophila* metamorphosis (B) [12,13,37]. Large black bands indicate the known protein–protein interactions.

A summary of the ecdysone regulatory cascade, with the 11 transcription factors known to act as classic early regulators during the onset of *Drosophila* metamorphosis, [12,13,37] is provided in Figure 8B. The gene expression profile of *E. granulosus* suggests that up-regulated genes play important roles in controlling and maintaining stage-specific features of the parasite during its life cycle [11]. It has been shown that bile acids (BAs) have a crucial role in the differentiation of PSCs into adult worms, and *E. granulosus* may express BA transporters and nuclear hormone receptors to stimulate the relevant pathways (Figure 8A) [11]. The mRNA expression of *EgHR3* was significantly increased under the stimulation of BA in the early stage of the development of the adult worm in *E. granulosus*, which suggests that BA can bind with the FXR/RXR to generate a BA-FXR/RXR complex, which then affects the transcriptional activity of EgHR3 to regulate adult development (Figure 8A) [38,39]. In insects, most effects of ecdysone are mediated through the heterodimeric ECR-USP [12]. ECR is clearly the invertebrate ortholog of FXR; USP being the invertebrate ortholog of RXR [35,36]. The FXR DNA-binding domain confers specific recognition of different DNA motifs called FXR response elements (FXRE). In *Drosophila*, the promoter region of the HR3 gene contains four putative ecdysone response elements (ECRE) and is activated by 20E through a binding of the ECR/USP complex to ECRE [40]. FXRE and ECRE are similar in terms of structure and function, so the BA-FXR/RXR complex may regulate the transcriptional activity of HR3 in *E. granulosus* (Figure 8A). In *Drosophila*, ecdysone binding to its receptors directly regulates the transcriptional activity of HR3 [40]; as the action model of ecdysone, the ecdysteroids in PSCs of *E. granulosus* can ultimately regulate the transcriptional activity of HR3 (Figure 8A). The orphan nuclear receptor HR3 is recognized as a central regulator in 20E-driven developmental switches during insect development and metamorphosis, and is responsible for directing timely shutdown of early genes regulated by a preceding 20E peak and a sequential activation of factors by a subsequent pulse of 20E [41]. We propose that a similar model of ecdysone action that occurs in insects is present in *E. granulosus* whereby *EgHR3* can regulate adult worm development, although the precise mechanisms involved require further investigation.

The silencing of *EgHR3* resulted in the death of 60% of PSCs, further indicating *EgHR3* is critically important for parasite survival and thereby represents a novel drug target for *E. granulosus* in the definitive and, perhaps also intermediate hosts, and a potential vaccine candidate against adult worms of *E. granulosus* in dogs.
